# Glucosylceramide synthase maintains influenza virus entry and infection

**DOI:** 10.1371/journal.pone.0228735

**Published:** 2020-02-07

**Authors:** Kelly Drews, Michael P. Calgi, William Casey Harrison, Camille M. Drews, Pedro Costa-Pinheiro, Jeremy Joseph Porter Shaw, Kendra A. Jobe, John D. Han, Todd E. Fox, Judith M. White, Mark Kester

**Affiliations:** 1 Department of Pathology, University of Virginia, Charlottesville, Virginia, United States of America; 2 Department of Biomedical Engineering, University of Virginia, Charlottesville, Virginia, United States of America; 3 Department of Environmental Sciences, University of Virginia, Charlottesville, Virginia, United States of America; 4 Department of Biology, University of Virginia, Charlottesville, Virginia, United States of America; 5 Department of Pharmacology, University of Virginia, Charlottesville, Virginia; 6 Department of Cell Biology, University of Virginia, Charlottesville, Virginia, United States of America; 7 Department of Microbiology, University of Virginia, Charlottesville, Virginia, United States of America; Deutsches Primatenzentrum GmbH - Leibniz-Institut fur Primatenforschung, GERMANY

## Abstract

Influenza virus is an enveloped virus wrapped in a lipid bilayer derived from the host cell plasma membrane. Infection by influenza virus is dependent on these host cell lipids, which include sphingolipids. Here we examined the role of the sphingolipid, glucosylceramide, in influenza virus infection by knocking out the enzyme responsible for its synthesis, glucosylceramide synthase (UGCG). We observed diminished influenza virus infection in HEK 293 and A549 UGCG knockout cells and demonstrated that this is attributed to impaired viral entry. We also observed that entry mediated by the glycoproteins of other enveloped viruses that enter cells by endocytosis is also impaired in UGCG knockout cells, suggesting a broader role for UGCG in viral entry by endocytosis.

## Introduction

Influenza A virus is the causative agent of influenza respiratory disease and is responsible for infecting between three and five million people worldwide each year. In 1918, an influenza pandemic resulted in one of the deadliest disease outbreaks in human history, killing an estimated 50 million people [1]. While a vaccine against influenza virus is produced annually, antigenic shift may result in influenza strains that circumvent vaccine efficacy and result in worldwide pandemics, such as the 2009 H1N1 pandemic [2].

A negative sense RNA virus belonging to the family *Orthomyxoviridae*, influenza virus is an endosome-entering enveloped virus that is encapsulated in a lipid membrane derived from its host cell. Inserted in the lipid envelope are two glycoproteins of influenza virus: hemagglutinin (HA) and neuraminidase (NA). During infection, influenza virus first binds to cell surface receptors via the HA protein and the virus is then internalized into an endosome. As the endosome acidifies to a pH of ~5.0–5.7, HA undergoes conformational changes that lead to fusion between the viral membrane and the endosomal membrane [3–7]. Upon successful viral fusion, the influenza virus genome is released into the cytoplasm and transported into the nucleus to undergo replication [8,9].

**Fig 1:**
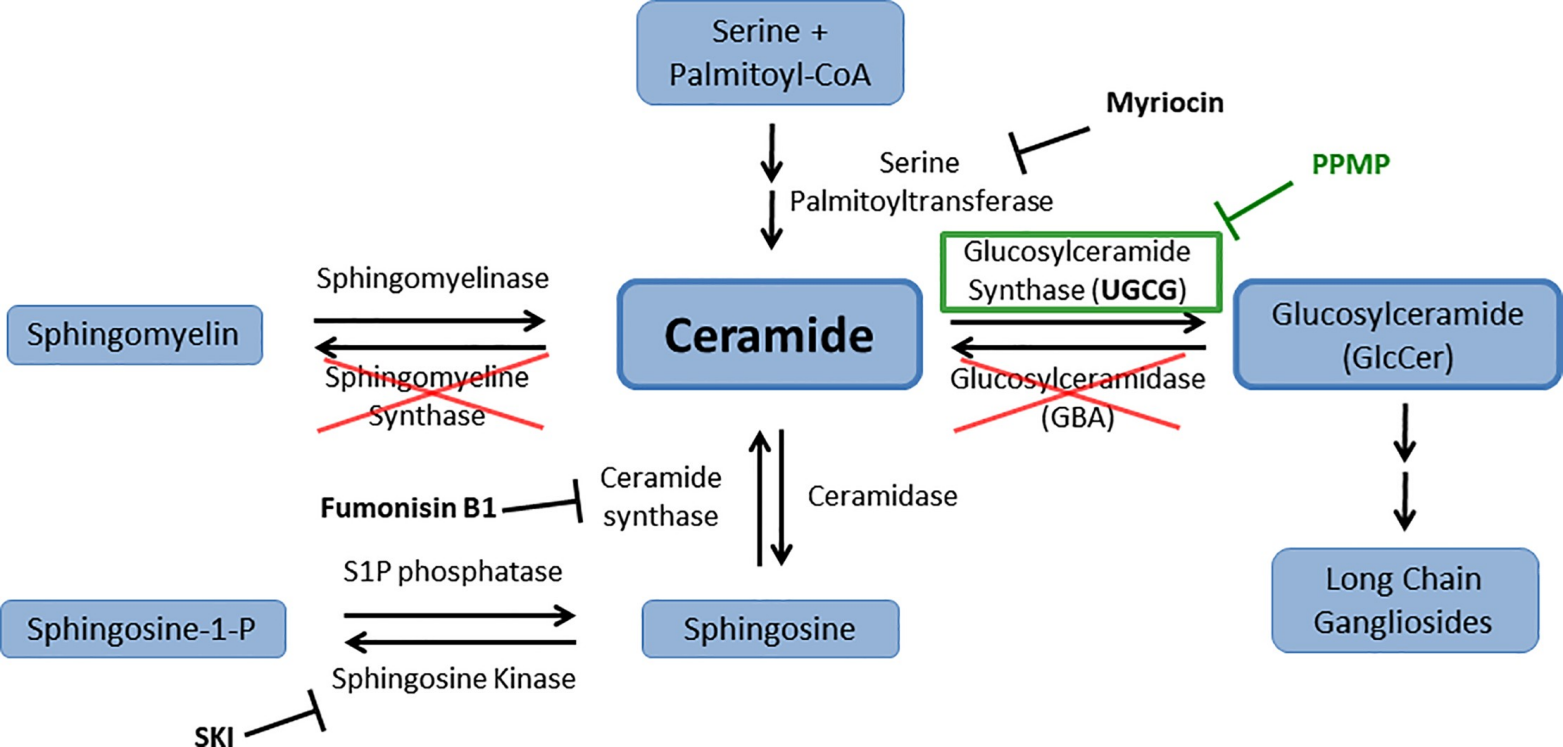
Sphingolipids and influenza virus infections. Several studies have demonstrated that inhibition of distinct enzymes in the sphingolipid pathway results in alterations to influenza virus infection levels. Pharmacological inhibition of sphingomyelin synthesis (through serine palmitoyltransferase) and sphingosine kinase, as well as genetic ablation of sphingomyelin synthase and glucosylceramidase (red Xs) led to decreased influenza virus infection [23–25,34]. Conversely, reductions in ceramide synthesis through inhibition of ceramide synthase led to an increase in influenza virus replication [22]. In this study we tested the role of UGCG (green box) in influenza virus infection by using the pharmacological inhibitor PPMP as well as by knocking out the gene encoding for UGCG enzyme expression in two cell lines.

The lipid membrane of influenza virus is enriched in sphingolipids, a major class of signaling molecules characterized by their sphingoid backbone [10]. Sphingolipids have been studied in the context of numerous viruses, and have been found to be critical to all stages of viral life cycles, including virus binding [11–14], entry [15–17], replication [18,19], and new particle release [20]. Several studies have investigated the role of specific sphingolipids in influenza virus infection, including sphingomyelin [21], ceramide [22], and sphingosine-1-phosphate [23,24] (**Fig 1**). Most recently, we determined that deletion of glucosylceramidase (GBA) leads to reduced influenza virus entry and impaired cellular endocytosis [25]. Loss of GBA led to increases in the amount of the glycosphingolipid, glucosylceramide, and delayed trafficking of influenza virus to late endosomes/lysosomes. Taken together, these studies suggest a need to further understand the regulation of glycosphingolipids in viral-host interactions.

Sphingolipid metabolism involves numerous enzymes and intermediary lipids, which predominantly shuttle through ceramide as a main hub [26] (**Fig 1**). Upon addition of a glucose molecule by glucosylceramide synthase (UGCG), ceramide is converted into the glycosphingolipid glucosylceramide (GlcCer), a pro-survival signaling molecule and a precursor lipid for higher order gangliosides [27]. GlcCer is a relatively understudied sphingolipid in the context of viral infections, as most research focuses on sphingomyelin, the far more abundant sphingolipid found primarily in plasma membranes. [11,15,16,28–30]. In addition to our study on GBA and GlcCer in influenza virus infection, a recent study explored the role for UGCG and GlcCer in bunyavirus infections and found that RNAi and pharmacological inhibition of UGCG led to loss of bunyavirus entry [17]. However, to our knowledge UGCG has never been studied in the context of influenza virus. Here we used CRISPR/Cas9 to genetically knockout UGCG and thereby determine its role in influenza virus entry and infection. We found that UGCG knockout cells displayed a reduction in influenza virus infection and entry, as well as reductions in entry of other endosome-entering viruses.

## Results

### Glucosylceramide synthase regulates influenza virus infection

**Fig 2:**
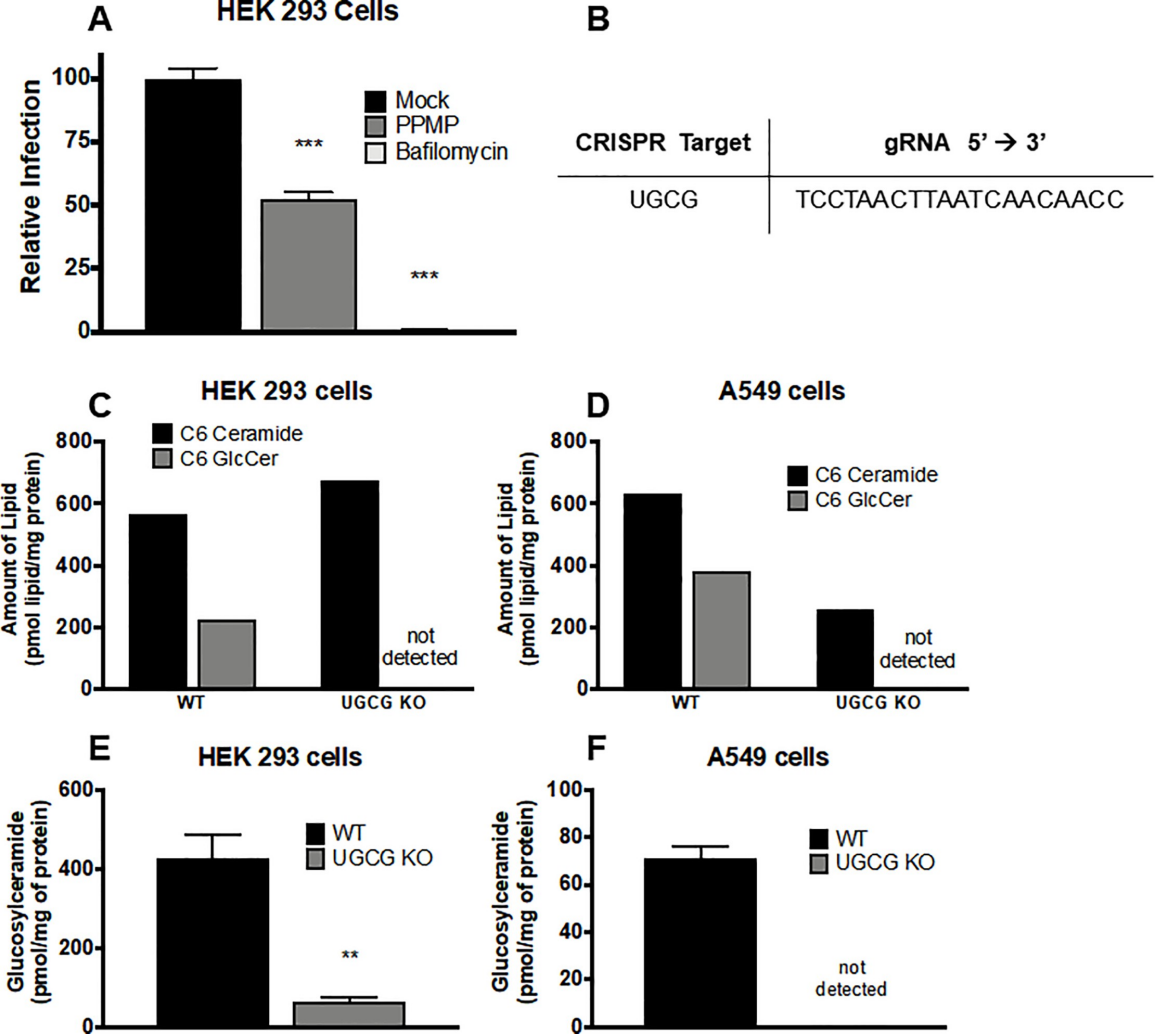
CRISPR/Cas9 mediated knockout of glucosylceramide synthase. **(A)** HEK 293 cells were pretreated with 20 μM PPMP (for 48 hours) or 100 nM bafilomycin (for 1 hour) and then infected with PR8 influenza virus encoding an NS1-GFP chimeric protein, in the presence of the indicated drug, for 18–24 hours (selected time points chosen after optimization). Cells were then lifted, fixed, and analyzed by flow cytometry for GFP expression. PPMP-treated samples exhibited a 50% reduction in GFP signal compared to WT, indicating a role for UGCG in influenza virus infection. Data represent the mean values of 4 biological replicates (each performed in triplicate) ± SE. **(B)** HEK 293 and A549 cells were transfected with plasmids encoding GFP as well as Cas9-sgRNA targeting UGCG. Cells were selected for GFP expression and single cell colonies were expanded and monitored for UGCG knockout as described in the Methods. **(C,D)** Selected cell clones (see **S1 Fig**) were assayed for UGCG activity by incubating cells with 5 μM C6-ceramide nanoliposome for 4 hours. Cells containing functional UGCG are able to convert C6-ceramide to C6-GlcCer, as seen in WT samples. HEK 293 and A549 UGCG KO cells displayed no C6-GlcCer, indicating a complete loss of UGCG activity. **(E,F)** Lipids from WT and KO cells were analyzed by mass spectrometry. In agreement with the measured enzyme activity **(C,D)**, levels of total basal endogenous GlcCer were significantly reduced in both HEK 293 and A549 KO cells as compared to WT. Data represent the mean ± SE (n = 6 samples). ** p<0.01 using a Mann-Whitney non-parametric test.

Previous studies demonstrated that loss of expression or inhibition of several sphingolipid-metabolizing enzymes leads to reduced influenza virus infection (**Fig 1**). We recently discovered that glucosylceramidase (GBA) is required for optimal influenza virus entry [25]. We thus hypothesized that glucosylceramide synthase (UGCG), which converts ceramide to glucosylceramide, may also play a role in the influenza virus life cycle (green box). When we treated target cells with PPMP, a broadly utilized inhibitor of UGCG [31–33], we observed a decrease in infection by PR8 influenza virus encoding NS1-GFP, as monitored by flow cytometry for GFP expression (**Fig 2A**). However, in order to ensure our results were not due to any off target effects of PPMP we sought to employ a genetic knockout of UGCG.

We therefore next employed CRISPR/Cas9 to knockout UGCG (see **Fig 2B** for gRNA sequence) in HEK 293 and A549 cells and determined the functional status of UGCG in putative knockout lines. HEK 293 cells were chosen for their ease of transfection and A549 cells were selected as a more physiologically relevant *in vitro* system for influenza virus research, as they were derived from human lung cells (and influenza virus is a respiratory pathogen). We screened potential UGCG KO clones by assaying for UGCG enzyme activity by incubating cells with C6-ceramide, a synthetic short-chain ceramide (data from the initial screen may be found in **S1A and S1B Fig**). Wild-type cells containing functional UGCG convert C6-ceramide to C6-GlcCer. However, in both HEK 293 and A549 UGCG KO cells, conversion of C6-ceramide to C6-GlcCer was not seen, indicating a full ablation of UGCG functional activity (**Fig 2C and 2D**). We next measured the endogenous basal (i.e. in uninfected cells) levels of GlcCer in WT and the chosen HEK293 and A549 KO cells, and determined that HEK 293 UGCG KO cells display significantly decreased GlcCer levels, and that GlcCer is undetectable in A549 UGCG KOs (**Fig 2E and 2F**). While, as expected, both cell types displayed reduced levels of GlcCer, ceramide levels were not correspondingly elevated (**S1 Table**), which may have resulted from “shunting” of ceramide to other downstream metabolites. Interestingly, ablation of UGCG activity in HEK 293 and A549 cells did not result in the same changes in downstream sphingolipid metabolic species between the two cell lines, as HEK 293 cells show an elevation in sphingosine-1-phosphate, while A549 cells display elevations in sphingomyelin (see Discussion). A full list of sphingolipid species regulated by glucosylceramide synthase can be found in **S1 Table**.

**Fig 3:**
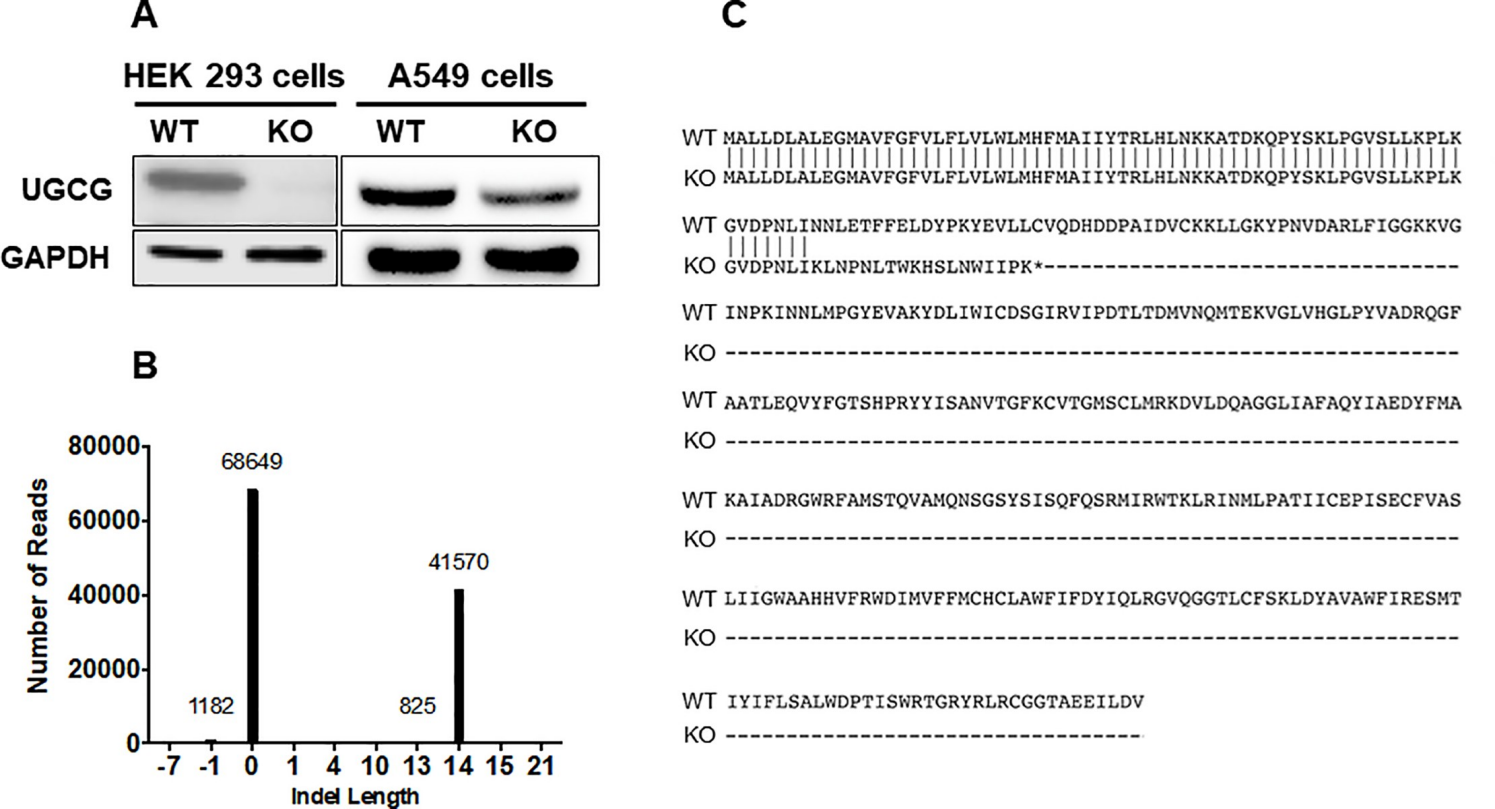
A549 UGCG KO cells exhibit haploinsufficiency. **(A)** Relative loss of UGCG expression was confirmed in HEK 293 cells by western blot analysis. In comparison, A549 UGCG KO cells (based on DNA analysis; see Methods) displayed only a reduced level (not an absence) of UGCG protein on Western blots. Since we detected no UGCG activity in these cells (**Fig 2D and 2F**), they are functionally null for UGCG and therefore haploinsufficient. **(B)** Next generation sequencing indicated the A549 UGCG KO line was heterozygous, containing one WT allele and one allele with a 14 base pair insertion in the gene encoding UGCG. This analysis supports the proposal that the loss of UGCG activity seen in **Fig 2D and 2F** is the result of haploinsufficiency. **(C)** Sequence analysis of the CRISPR-modified UGCG allele in A549 cells revealed a frameshift mutation beginning at the codon for amino acid N68 and terminating with an early stop codon at the position of C86.

The selected UCGC KO clonal cell lines were then assessed for loss of UGCG protein by western blotting (**Fig 3A**). Consistent with the results of lipid mass spectrometry (**Fig 2C**–**2F**), there was no detectable UGCG protein in HEK 293 UGCG KO cells. However, despite the indicated KO of enzyme activity **(Fig 2C**–**2F)**, western blots of A549 UGCG KO cells displayed a signal for UGCG protein, albeit in reduced amount compared to WT cells. To address this apparent conundrum, we performed next-generation sequencing to determine the exact genetic alterations that had occurred in the A549 UGCG KO cells. We determined that those cells displayed a heterozygous mutation (**Fig 3B**), with one allele altered by the CRISPR/Cas9 activity to contain a premature stop codon (**Fig 3C**) while the other allele remained unaltered. These findings suggest that the induced mutation (stop codon) resulted in haploinsufficiency, as the functional activity of UGCG was completely lost in A549 KO cells (**Fig 2C**–**2F**).

**Fig 4:**
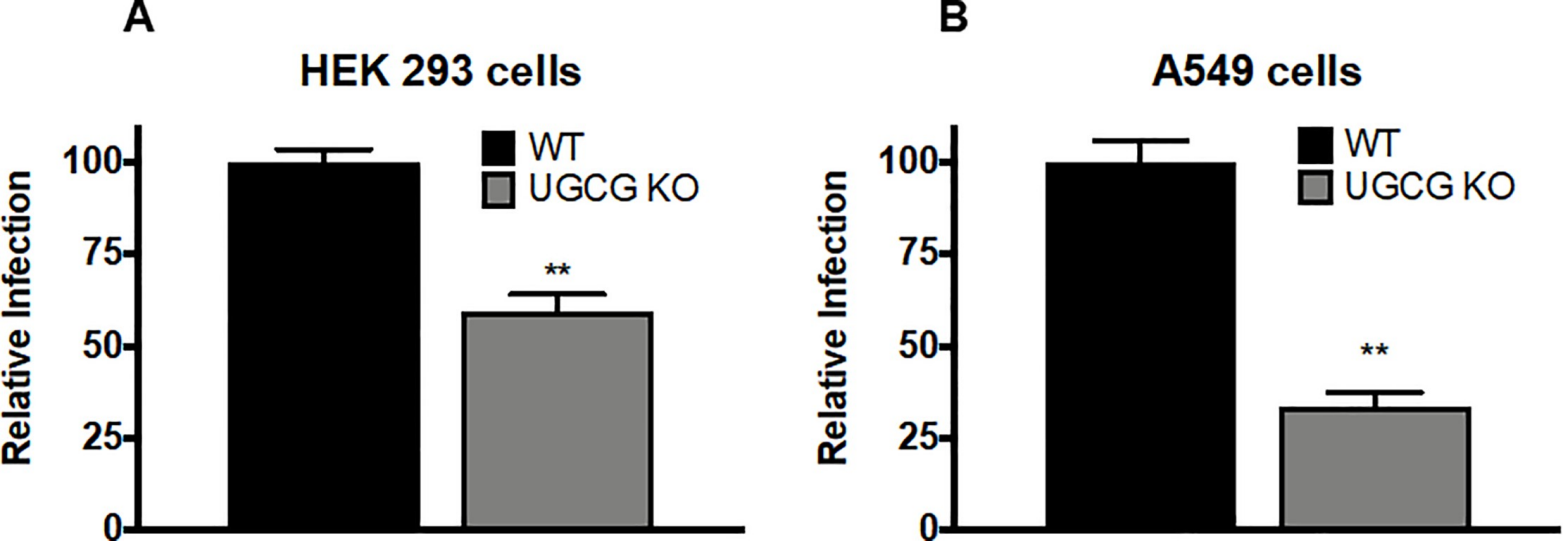
Glucosylceramide synthase regulates influenza virus reinfection. Cells were infected with influenza virus as in **Fig 2A**, and analyzed 18–24 hours later by flow cytometry. **(A)** HEK 293 UGCG KO cells exhibited an ~40% reduction in influenza virus infection as compared to WT. **(B)** A549 UGCG KO cells exhibited ~70% reduction in influenza virus infection as compared to WT. (mean ± SE; n = 6). ** p<0.01 using a Mann-Whitney non-parametric test.

Finally, to determine if knockouts of UGCG affect influenza virus infection, we used the GFP-encoding PR8 influenza virus as in Fig 2A. As seen in **Fig 4A and 4B**, influenza virus infection levels were decreased in both HEK 293 and A549 UGCG KO cells compared to WT cells. Together these data demonstrate that pharmacological and molecular disruption of cellular GlcCer synthesis leads to suppression of influenza virus infection.

### Glucosylceramide synthase regulates entry of influenza virus and other endocytosed viruses

**Fig 5:**
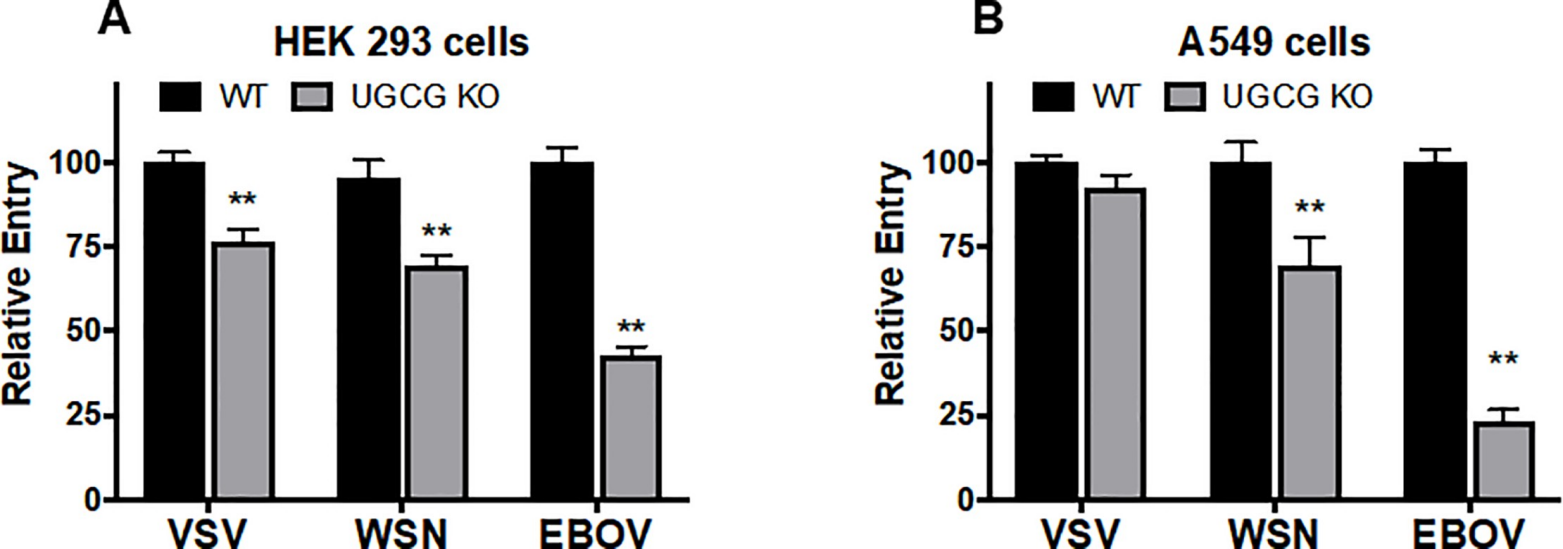
UGCG maintains optimal entry of VLPs bearing the glycoproteins of VSV, WSN influenza virus and EBOV. VLPs were generated on an influenza virus βlaM1 backbone with the indicated viral glycoprotein. VLPs were added to prechilled cells which were then centrifuged at 4° for 1 hour. Next the cells were incubated for 3 hours at 37°, and then for 1 hour at room temperature in the presence of the βlaM substrate CCF2. Cells were washed, stored in the dark at room temperature, and (the following day) harvested, fixed, and analyzed for β-lactamase activity via flow cytometry. **(A,B)** Entry by VLPs bearing VSV G was reduced in HEK 293 UGCG KO cells, but unaffected in A549 KO cells. WSN influenza virus glycoprotein-mediated entry was reduced in both KO cell lines, consistent with the findings in **Fig 4**. Entry mediated by the EBOV glycoprotein was reduced in both 293 and A549 UGCG KO cells, and to a greater extent than seen with VLPs bearing the glycoproteins from WSN or VSV (mean ± SE; n = 6). ** p<0.01 using a Mann-Whitney non-parametric test.

**Fig 6:**
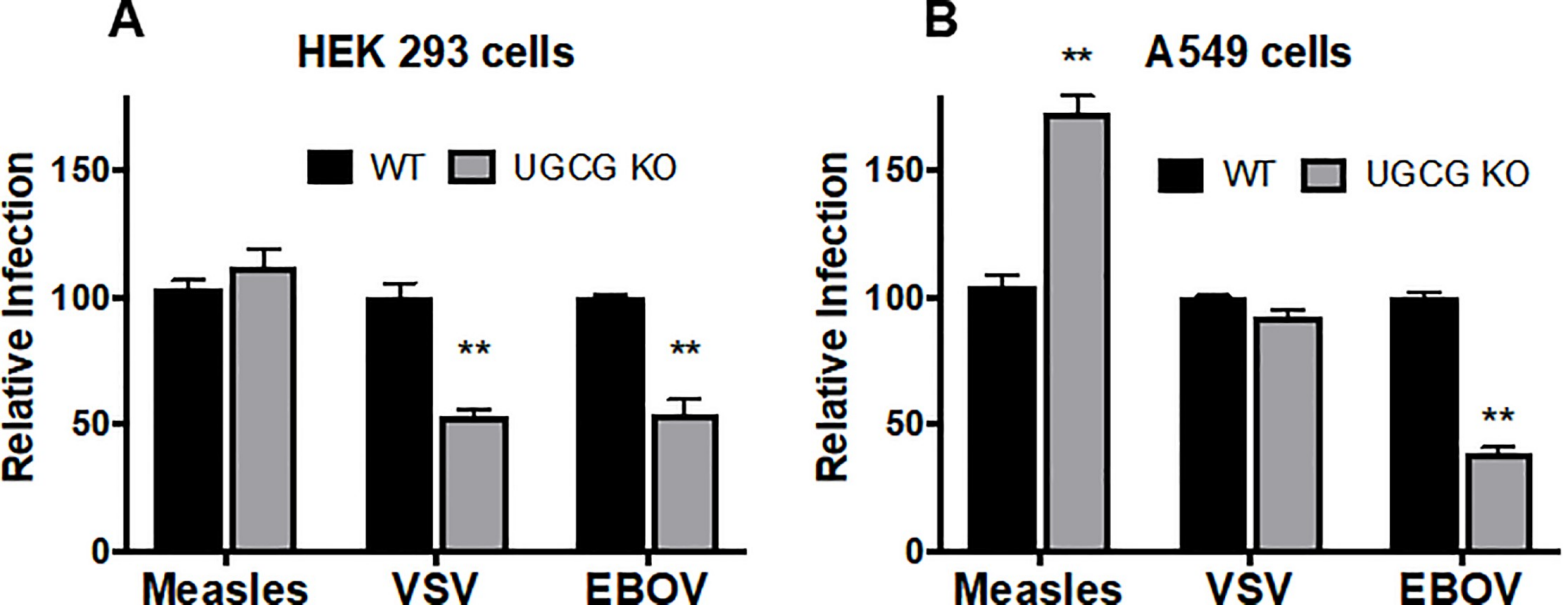
Effects of loss of UGCG on infections by VSV pseudoviruses bearing the glycoproteins of measles virus, VSV, and EBOV. Pseudoviruses were generated using a VSV helper virus encoding GFP and the indicated viral glycoprotein(s). Pseudoviruses were then adhered to prechilled cells assisted by centrifugation at 4°C for 1 hour. The cells were then washed, incubated at 37°C and, the following day, harvested, fixed, and analyzed for GFP expression by flow cytometry. **(A,B)** Infection by VSV pseudoviruses bearing the measles virus H and F proteins was unaffected in HEK 293 UGCG KO cells, and increased in the A549 UGCG KO cells. Infection by VSV pseudoviruses bearing the VSV glycoprotein was decreased in HEK 293 UGCG KO cells but unaffected in A549 UGCG KO cells, consistent with the findings in **Fig 5**. Infection by VSV pseudoviruses bearing the EBOV glycoprotein was decreased in both UGCG KO lines tested (mean ± SE; n = 6), also consistent with the results in **Fig 5**. ** p<0.01 using a Mann-Whitney non-parametric test.

We hypothesized that the reduction in influenza virus infection in UGCG KO cells was due to a reduction in influenza virus entry into the cells. To address this, we generated virus-like particles (VLPs) containing an influenza virus Matrix-1 (M1)-β-lactamase (β-lam) core and a membrane bearing the HA and NA glycoproteins of WSN influenza virus, which fuses with host endosomes at pH ~5.9–6.0 [35]. We extended the analysis to include influenza virus Matrix-1 VLPs displaying the glycoproteins of vesicular stomatitis virus (VSV), which fuses with host early endosomes (pH ~6.0), and Ebola virus (EBOV), which fuses with host endolysosomes (pH ~4.5–5.5) [36–38]. We determined that both WSN influenza virus HA/NA and EBOV GP Matrix-1 VLPs displayed reduced entry into both HEK 293 and A549 UGCG KO cells compared to WT cells. VSV G VLPs displayed reduced entry into HEK 293 UGCG KOs, but not into A549 UGCG KOs (**Fig 5A and 5B**), which may be due to the different tissue origins of these cells. To further explore the extent to which UGCG mediates viral infections, we employed a VSV pseudovirus system and examined how pseudoviruses bearing the glycoproteins of VSV, EBOV, and measles (a virus that employs its H and F proteins to fuse at the plasma membrane) infect UGCG KO and WT cells. As seen in **Fig 6A and 6B**, and similar to the results seen with influenza virus Matrix-1 VLPs (**Fig 5**), VSV G-mediated pseudovirus infection was reduced in HEK 293 UGCG KOs, but unaffected in A549 UGCG KOs; EBOV GP pseudovirus infection was reduced in both UGCG KO cell lines tested. In contrast, measles H/F-mediated pseudovirus infection was not inhibited in HEK 293 UGCG KOs, and was even increased in A549 UGCG KO compared to WT cells. These results indicate a role for UGCG in influenza virus entry, and suggest a similar function in the entry of other endosome-entering viruses, particularly ones that enter late in the endocytic pathway.

## Discussion

Previously, we found that deleting GBA1, the enzyme that converts GlcCer (the primary product of UGCG enzymatic activity) to ceramide (Fig 1) increases GlcCer levels and impairs endosome trafficking and influenza virus entry [25]. Here we found that knocking out UGCG decreases GlcCer levels (in both HEK 293 and A549 cells), impairing the entry of endosome-entering viruses. This could potentially suggest that specific optimal, highly-regulated amounts of GlcCer (as opposed to a threshold amount) may be needed for optimal endocytic trafficking. These findings are consistent with a previous study that found that inhibition of either UGCG or GBA led to mistargeting of glycosphingolipids from the Golgi to lysosomes [39]. We propose a preliminary hypothesis that a homeostatic concentration of sphingolipids is necessary to maintain optimal infection of influenza virus, and that disruption of the balance between the production and consumptions of GlcCer could be responsible for the reduction in influenza virus infection observed in all of the GBA and UGCG KO cell lines tested. In support of the proposed GlcCer homeostasis model, examination of the lipid profiles of GBA and UGCG KO cells yielded one consistent theme: of all of the lipids analyzed, only GlcCer was consistently increased (in the case of GBA KOs) or decreased (in the case of UGCG KOs). However, these findings must be further examined to test if there is a clear connection between homeostatic levels of GlcCer and optimal cellular endocytosis.

The importance of maintaining the right balance of key molecules has been shown in several other cellular systems. For instance, tightly regulated signaling pathways often require a homeostatic state to maintain proper cell function (such as Notch signaling), and both up- and down-regulation of specific proteins has been shown to cause constitutive Akt signaling [40,41]. In addition, GlcCer serves as the foundational lipid for a variety of higher order glycolipids, including lactosylceramide, GM3, and GMD3. Disruptions in GlcCer levels may result in disruptions in the homeostasis of any number of gangliosides, many of which have been implicated in a number of cellular functions, including maintenance of lipid rafts [42]. For a complete analysis of the effects of knocking out GlcCer metabolizing enzymes on influenza virus infection these long chain gangliosides must be considered as well as the numerous sphingolipids listed in **S1 Table,** and these questions remain an avenue for future research.

We hypothesize that because ceramide is a pro-apoptotic molecule [26], cells limit any build-up of this bioactive lipid by converting it to either sphingomyelin or sphingosine/S1P. In HEK 293 UGCG KO cells sphingomyelin levels are comparable to those in WT cells, but sphingosine-1-phosphate levels increase 14-fold, indicating that blocking GlcCer production by removing UGCG may shunt sphingolipid production to sphingosine-1-phosphate. Interestingly, sphingosine-1-phosphate was recently shown to heighten cellular susceptibility to influenza virus infection and as such may have masked part of the effect of knocking out UGCG on influenza virus entry [23,24]. A549 UGCG KOs display a modest increase in sphingosine-1-phosphate levels as well as a larger increase in sphingomyelin, the most prevalent sphingolipid found in cells and predominantly localized to the plasma membrane [43]. We hypothesize that the elevated sphingomyelin levels in A549 UGCG KO cells contribute to the increase in entry mediated by the glycoproteins of measles virus seen in A549 UGCG KO cells (**Fig 6B)**, as measles virus enters cells by fusion with the plasma-membrane [44]. Additional studies into altered sphingolipid metabolism are required to test the proposed compensatory mechanisms that maintain ceramide levels in the context of UGCG ablation. We further hypothesize that the differences in sphingolipid compensation between HEK 293 and A549 UGCG KO cells may be due to differences in their basal levels of various sphingolipid enzymes, such as sphingomyelin synthase or sphingosine kinase. It is important to note that any perturbations of sphingolipid levels may have an effect on influenza virus infection and therefore future investigation into the expression levels of these critical mediators of sphingolipid metabolism will be necessary to better understand the implications of our data for sphingolipid biology and viral entry.

While we postulate that the lack of glycosphingolipids contributes to diminished influenza virus entry, we cannot rule out that compensatory ceramide metabolites like sphingomyelin or S1P contribute to this defect. It is important to note that the data in **S1 Table** are a measurement of the cellular sphingolipid content at one time point after CRISPR/Cas9 mediated knock out of UGCG. To further investigate the observed changes in lipid species upon knocking out UGCG, measurements of lipid flux using isotopically-labeled precursors should be evaluated. In addition, a time-course experiment using inhibitors of UGCG may help to clarify whether UGCG’s role in influenza virus infection is a result of its direct metabolites (such as glucosylceramide) or compensatory downstream products.

In summary, the findings presented in this study demonstrate a previously undiscovered role for UGCG in influenza virus entry. By both pharmacological inhibition and genetic ablation, cells deficient in UGCG activity were found to display reduced entry and infection by influenza virus as well as entry mediated by the glycoproteins of other endosome-entering viruses including EBOV. We hypothesize that these reductions in viral entry are due to a general impediment of normal endocytic trafficking, based on previous studies of GlcCer metabolizing enzymes by both ourselves [25] and the Bates laboratory [45]. We showed that GBA is needed at a post binding and internalization step for proper trafficking of both EGF and influenza virus particles to late endosomes [25]. Bates and coworkers demonstrated a role for UGCG in infections by specific bunyaviruses at a post-internalization step and importantly, that study ruled out the need for UGCG for virus binding or internalization [45]. Their hypothesis that UGCG may be involved in endosome trafficking or virus-endosome fusion is in concordance with the findings presented in this manuscript [17]. These collective results suggest that UGCG provides an intriguing candidate for further study in the context of viral entry and as a potential novel target for future influenza therapies.

## Materials and methods

### Cells

BHK-21 (baby hamster kidney; ATCC CCL-10), A549 (human lung carcinoma; ATCC CCL-185), HEK 293T/17 (ATCC CRL-11268), and HEK 293 (human embryonic kidney; ATCC CRL-1573) cells were grown in Dulbecco modified Eagle medium (DMEM) supplemented with 10% fetal bovine serum (FBS), 1% antibiotic/antimycotic, 1% L-glutamine, and 1% sodium pyruvate at 37°C (Gibco Life Technologies) in a 5% CO_2_ incubator.

### CRISPR/Cas9 gene editing

The CRISPR design tool from the Zhang laboratory (available at crispr.mit.edu) was used to select a gRNA targeting UGCG. Selected gRNA was cloned into a Cas9-sgRNA (Addgene plasmid# 68463, deposited by Su-Chun Zhang) using BbsI. Following cotransfection of the resulting plasmid and one encoding GFP (plasmid backbone: pLEGFP) into HEK 293 and A549 cells, single GFP-expressing cells were isolated using an Influx flow cytometer and expanded. All colonies were analyzed for shifts in UGCG DNA fragment size by PCR, and then 5–10 colonies per cell line were analyzed by mass spectrometry (for enzyme activity and sphingolipid content) and western blotting (for UGCG protein). No difference in growth rate was seen for WT and UGCG 293/A549 KO cells over the time period analyzed (4 days). Growth rates were measured by MTS assay over a period of four days.

### Inhibitors and other reagents

PPMP (Cat# P4194) and Bafilomycin A1 (Cat# B1793) were purchased from Sigma-Aldrich. BbsI was purchased from New England Biolabs (Cat# R0539S).

### Influenza viruses, influenza virus VLPs, and VSV pseudoviruses

Purified PR8 IAV was obtained from Charles River Laboratories. PR8 NS-GFP was provided by Dr. Thomas Bracciale at the University of Virginia [46]. All influenza viruses were grown in embryonated chicken eggs, thereby cleaving HA_0_ to its infectious form (HA1-S-S-HA2) [47,48].

VSV pseudoviruses encoding GFP were produced in BHK-21 cells plated at 5x10^5^ cells/dish in forty 10cm^2^ dishes. The cells were transfected (when ~75–80% confluent) with plasmids encoding, as indicated, VSV-G (plasmid backbone: pCAGGS), EBOV-GPΔ (plasmid backbone: VRC6002), or Measles F and H (plasmid backbone: PCXN2), with polyethylenimine (PEI; Polysciences, Inc Cat# 23966). Measles F and H (Edmonston strain) plasmids were generously provided by Dr. Yusuke Yanagi of Kyushu University This strain of measles virus was reported to use CD46 as its receptor on non-lymphoid cells [49]. 24 hours later, cells were infected with pre-titered VSV-ΔG helper virus encoding GFP (from a plaque eluate) at 37°C for 1 hr, washed extensively with PBS, and then incubated overnight at 37°C in growth medium. The next day cell supernatants containing budded pseudoviruses were collected, the debris cleared by two rounds of centrifugation (1360 x g/10 min), and then concentrated ~50-fold using a Viva-Spin 20 300kDa concentrator. Finally, the concentrated pseudovirus was centrifuged through a 20% sucrose cushion (in HEPES-MES [HM] buffer: 20 mM HEPES, 20 mM MES, 130 mM NaCl, pH 7.4) in an SW28 rotor for 2 hours at 112,398 x g at 4°C, and then resuspended in 10% sucrose-HM. Pseudovirus stocks were stored at -80°C.

VSV-ΔG helper virus was produced as described previously [50]. In brief, BHK-21 cells plated in five 10cm^2^ dishes (each with 5x10^5^ cells) at ~75–80% confluency were transfected with 12 μg (per dish) of a plasmid encoding VSV-G using PEI. The next day cells were infected with 40 μl of VSV-GFP plaque eluate (3.39 x 10^8^ infectious units/mL) for 1 h at 37°C in serum-free media. Cells were washed extensively with PBS and incubated in complete media overnight at 37°C. ~24 hours later supernatants containing helper virus were collected, centrifuged at 1070 x g for 10 min two times to clear debris, and stored at -80°C.

Influenza virus M1-VLPs were made by transfecting HEK 293T/17 cells in each of 5 10cm^2^ dishes (each with 1x10^6^ cells) in complete media without antibiotic/antimycotic using plasmids encoding βlaM1 and either EBOV-GPΔ, VSV-G, or WSN HA + WSN NA (plasmid backbone: pCAGGS) using PEI. WSN is an H1N1 strain of influenza virus that is trypsin-independent *in vitro* [51]. The βlaM1 plasmid was provided by Dr. Adolfo Garcia-Sastre and the NIAID Centers of Excellence for Influenza Research and Surveillance (CEIRS) program [52]. Media containing VLPs was harvested at 24 and 48 hours post transfection, pooled, and centrifuged two times to clear debris. The VLPs were then pelleted through a 20% sucrose cushion in HM buffer using an SW28 rotor at 4°C at 112,398 x g for 2 hours. Finally, VLPs were resuspended in 10% sucrose-HM and were stored at -80°C.

### IAV reporter infection assay

Cells were seeded at a density of 3x10^4^ cells per well in 96 well plates. ~24 hours later the cells were incubated with PR8 influenza virus encoding GFP fused to the N-terminus of NS1 at an MOI of ~1 in growth medium without trypsin or FBS and centrifuged at 4° for 1 hour at 250 x g. The cells were then incubated at 37°C for ~16–18 hours, lifted with trypsin, fixed in 4% paraformaldehyde (PFAM), and assayed on an Attune NxT flow cytometer for GFP signal. Uninfected cells were used to set a background value for GFP. For infection assays in the presence of inhibitors, cells were pre-treated with 20 μM PPMP for 48 hours or 100 nM Bafilomycin for 1 hour before adding PR8 in the presence of the indicated inhibitor. All values were normalized to mock infected cells.

### Influenza virus M1-VLP entry assay

Cells were seeded at a density of 3x10^4^ cells per well in 96 well plates. ~24 hours later the cells were incubated with influenza virus M1-VLPs diluted in Opti-MEM I (OMEM) and centrifuged at 4°C for 1 hour at 250 x g. The cells were incubated for 3 hours at 37°C and then the βlam substrate CCF2-AM (Invitrogen, cat# K1032) was added in loading buffer (phenol red-free DMEM containing 20 mM (A549) or 5 mM (HEK 293) probenecid (MP Biomedicals, cat# 156370)), 25 mM HEPES, 2 mM L-glutamine, 200 nM bafilomycin) and incubated for ~1 hour at room temperature. The cells were then washed with PBS and incubated overnight in the dark at room temperature in loading buffer containing 10% FBS. 18–24 hours later, the cells were lifted with trypsin, fixed in 4% PFAM, and analyzed on an Attune NxT flow cytometer for CCF2-AM cleavage (marked by a color shift from green (518nm) to blue (447nm)).

### VSV pseudovirus infection assay

3x10^4^ cells were seeded per well in 96 well plates. 24 hours later the cells were incubated with VSV pseudoviruses in Opti-MEM I (OMEM) followed by centrifugation at 4°C for 1 hour at 250 x g. the cells were washed and then incubated in growth medium at 37°C for 18–24 hours after which they were lifted, fixed, and analyzed for GFP expression on an Attune NxT flow cytometer.

### Western blotting

Cell samples were lysed in PBS with 5 mM EDTA, 1 mM sodium vanadate (Sigma; Cat# S6508) and 1% SDS. Proteins in the cell lysates were separated via SDS-PAGE and transferred to PVDF membranes. The membranes were then probed with the indicated primary antibodies followed by secondary antibodies coupled to horseradish peroxidase. Visualization of signal was achieved with a chemiluminescent HRP substrate and images were captured with an Alpha-Innotech Fluorchem detector.

### Antibodies

Antibodies were purchased from the following sources: anti-GAPDH (14C10), Cell Signaling Technology (Cat# 3683); anti-UGCG (M03), Abnova (Cat# H00007357).

### Preparation of C6 ceramide nanoliposomes

C6 ceramide nanoliposomes were prepared as described previously [53]. Briefly, 1,2-distearoyl-sn-glycero-3-phosphocholine,1,2-distearoyl-sn-glycero-3-phosphoethanolamine-*N*-[methoxy polyethyleneglycol-2000], 1,2-dioleoyl-*sn*-glycero-3-phosphoethanolamine, *N*-octanoyl-sphingosine-1-[succinyl(methoxypolyethylene glycol-750)] (PEG(750)-C_8_), and *N*-hexanoyl-d-erythro-sphingosine (C_6_-ceramide) were combined in chloroform at a molar ratio of 3.75:3:1.75:0.75:0.75. The lipid mixture was dried and then rehydrated followed by sonication and extraction through a 100 nm polycarbonate membrane.

### Lipid mass spectrometry and enzyme activity assay

For the UGCG enzyme activity assay, cells were incubated with 5 μM C6 ceramide nanoliposomes (~100 nm) for 4 hours, collected, and then subjected to lipid extraction as described previously (45). Lipid species were then analyzed, as described previously, on an Acquity I-Class/Xevo TQ-S system [54]. Internal standards were compared to mass spectrometry peaks and all data are represented as pmol of lipid/mg of protein.

## Supporting information

S1 Fig Screening and analysis of putative UGCG KO clones. **(A and B)** Putative KO clones from both HEK293 and A549 cells were screened (n = 1) by determining their ability to convert exogenously added C6 ceramide to C6 GlcCer, which was assayed by lipid mass spectroscopy. The clones that exhibited the greatest reduction in UGCG activity were selected for further experiments: clone B29 for HEK293 and clone A11 for A549 cells. **(C and D)** The chosen KO cell lines were analyzed to determine the effect of knocking out UGCG on cell growth rates to ensure that any experimental findings were not due to underlying differences in cell growth. (n = 1, performed in triplicate).
(TIF)

S1 Table Full basal sphingolipid profiles from UGCG knockout cells. Sphingosine, glucosylceramide, sphingomyelin, sphingosine-1-phosphate, and ceramide in uninfected KO and WT cells were analyzed by liquid chromatography-mass spectrometry. The data represent the averages from five biological replicates and are represented as pmol lipid/mg of protein.
(TIF)
