# Supplementary figures and images for: Glucosylceramide synthase maintains influenza virus entry and infection
Source: PLoS One. 2020 Feb 7;15(2):e0228735. doi: 10.1371/journal.pone.0228735 (PMC7006932; doi:10.1371/journal.pone.0228735)

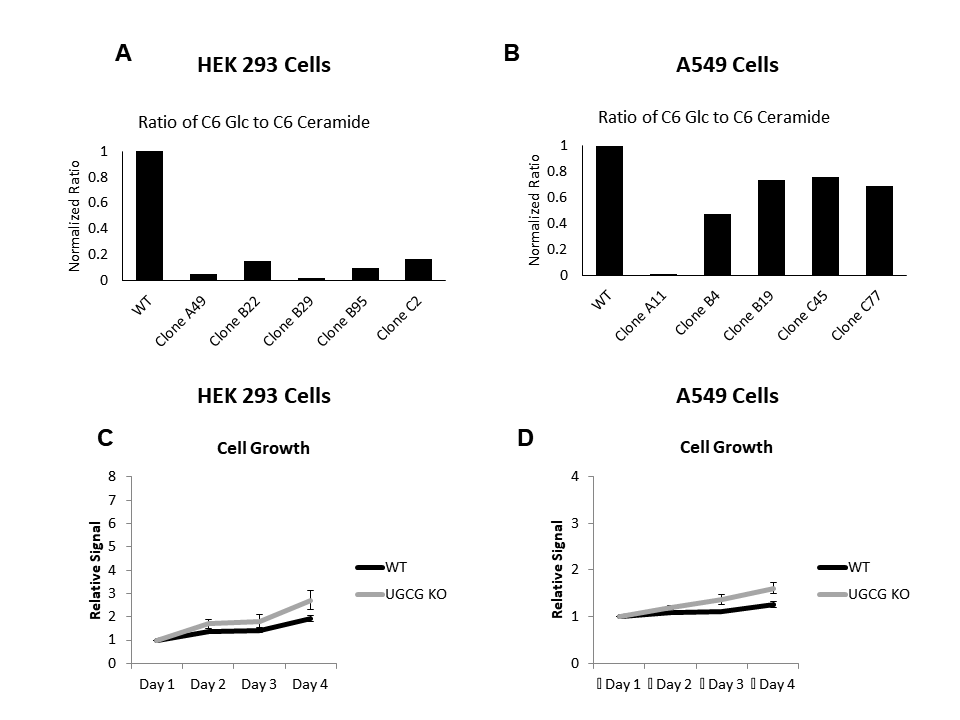

Supplement: S1 Fig — (A and B) Putative KO clones from both HEK293 and A549 cells were screened (n = 1) by determining their ability to convert exogenously added C6 ceramide to C6 GlcCer, which was assayed by lipid mass spectroscopy. The clones that exhibited the greatest reduction in UGCG activity were selected for further experiments: clone B29 for HEK293 and clone A11 for A549 cells. (C and D) The chosen KO cell lines were analyzed to determine the effect of knocking out UGCG on cell growth rates to ensure that any experimental findings were not due to underlying differences in cell growth. (n = 1, performed in triplicate). (TIF) [file pone.0228735.s001.TIF]

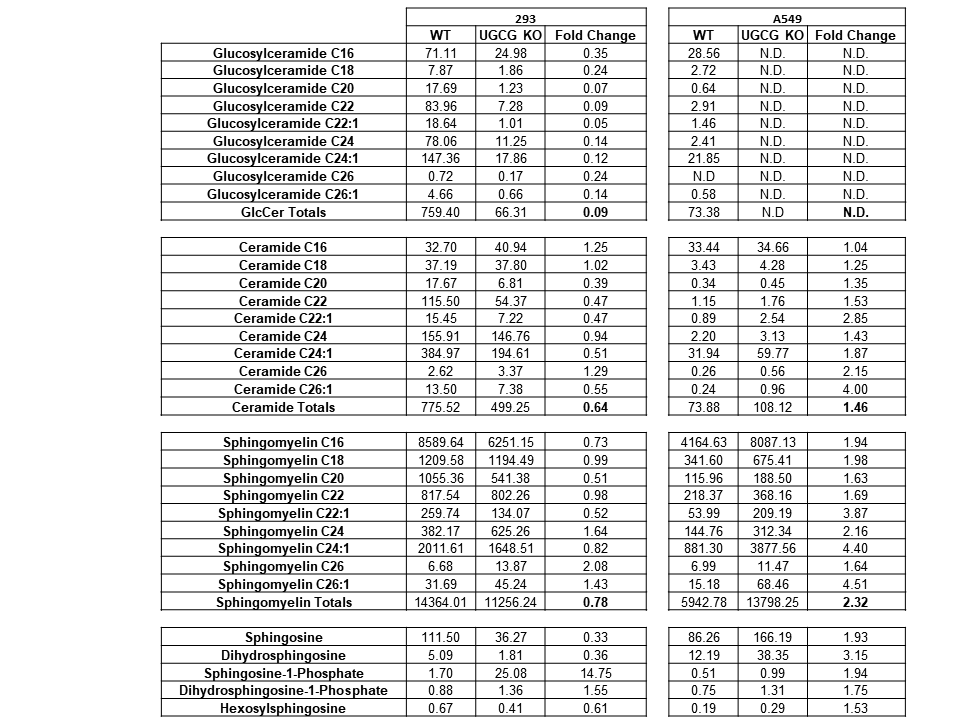

Supplement: S1 Table — Sphingosine, glucosylceramide, sphingomyelin, sphingosine-1-phosphate, and ceramide in uninfected KO and WT cells were analyzed by liquid chromatography-mass spectrometry. The data represent the averages from five biological replicates and are represented as pmol lipid/mg of protein. (TIF) [file pone.0228735.s002.TIF]
